# Impact of perinatal factors on meconium aspiration syndrome in full-term newborns and the construction of a column chart prediction model: An observational study

**DOI:** 10.1097/MD.0000000000038279

**Published:** 2024-05-17

**Authors:** Chun-Yu Wang, Chen Ling, Juan-Juan Yang, Li-Sha Guan, Xiao-Qing Wang

**Affiliations:** a Obstetrical Department of Suzhou Ninth People’s Hospital, Suzhou, Jiangsu, China.

**Keywords:** influencing factors, meconium aspiration syndrome, newborns, prediction model, umbilical blood gas analysis

## Abstract

To explore the influence of perinatal-related factors on meconium aspiration syndrome (MAS) in full-term neonates and construct a nomogram prediction model for risk stratification of neonatal MAS and adoption of preventive measures. A total of 424 newborns and their mothers who were regularly examined at our hospital between January 2020 and December 2023 who had meconium-contaminated amniotic fluid during delivery were retrospectively selected as participants. Neonates were divided into MAS and non-MAS groups based on whether MAS occurred within 3 days after birth. Data from the 2 groups were analyzed, and factors influencing MAS were screened using multivariate logistic regression analysis. The R3.4.3 software was used to construct a nomogram prediction model for neonatal MAS risk. Receiver operating characteristic (ROC) curve analysis and the Hosmer–Lemeshow goodness-of-fit test were used to evaluate the performance of the model, and its clinical effectiveness was evaluated using a decision curve. Among the 424 neonates with meconium-stained amniotic fluid, 51 developed MAS within 3 days of birth (12.03%). Multivariate logistic regression analysis showed that a low amniotic fluid index before delivery (OR* *= 2.862, *P *= .019), advanced gestational age (OR* *= 0.526, *P *= .034), cesarean section (OR* *= 2.650, *P *= .013), severe amniotic fluid contamination (OR* *= 4.199, *P *= .002), low umbilical cord blood pH (OR* *= 2.938, *P *= .011), and low neonatal Apgar 1-min score (OR* *= 3.133, *P *= .006) were influencing factors of MAS in full-term neonates. Based on the above indicators, a nomogram prediction model for MAS risk of full-term newborns was constructed. The area under the ROC curve of the model was 0.931. The model was also tested for goodness-of-fit deviation (*χ*^2^ = 3.465, *P *= .903). Decision curve analysis found that the model was clinically effective in predicting the net benefit of MAS risk in neonates with meconium-stained amniotic fluid. The construction of a column chart prediction model for neonatal MAS risk based on prenatal amniotic fluid index, gestational age, delivery method, amniotic fluid contamination level, newborn umbilical blood pH value, and Apgar 1-min score has a certain application value.

## 1. Introduction

Meconium aspiration syndrome (MAS) refers to the inhalation of amniotic fluid mixed with meconium by the fetus in the uterus or during delivery. MAS is a respiratory disease that causes airway obstruction and lung inflammation.^[1]^ Therefore, the detection of meconium-contaminated amniotic fluid is necessary to diagnose neonatal MAS.^[2]^ The main pathological features of MAS are mechanical obstruction of the respiratory tract and chemical inflammation of the lung tissue,^[3,4]^ that present as difficulty in breathing and worsening of hypoxia and ischemia shortly after birth. The incidence of MAS is higher in full-term and expired infants.^[5,6]^ Following onset of MAS, it will evolve as the condition progresses, causing damage to various organ systems throughout the body that further threatens the physical health of newborns.^[7]^ Therefore, it is necessary to strengthen research on the mechanisms of neonatal MAS to facilitate clinical measures to reduce its incidence. In the era of precision medicine, models use clinical information to demonstrate the risk of certain diseases. However, current research on MAS in full-term newborns mainly focuses on analyzing influencing factors, and research on predictive models for the risk of MAS in this population is lacking. This study analyzed the clinical data of full-term pregnant women who underwent regular prenatal examinations and their newborns to identify factors influencing MAS in full-term newborns and construct corresponding predictive models, which were expressed in the form of a nomogram, to objectively present the variables of the prediction model, their corresponding scores, and the predicted event occurrence rate. The results should provide a reference for the clinical identification of the risk of neonatal MAS in full-term pregnancy and allow for the adoption of early preventive measures.

## 2. Materials and methods

### 2.1. Research object

In this retrospective study, we selected newborns and their mothers who underwent regular prenatal examinations at Suzhou Ninth People’s Hospital between January 2020 and December 2023 and had meconium-contaminated amniotic fluid during delivery. The inclusion criteria were mothers who met the diagnostic criteria for meconium-contaminated amniotic fluid, which is a brownish-yellow viscous fluid with small amounts of granular meconium as well as fecal staining of the umbilical cord and fetus;^[8]^ provided consent for study inclusion; underwent full-term (gestational weeks ≥ 37 weeks) delivery; underwent natural conception; and had a singleton pregnancy. The exclusion criteria were as follows: pregnant women who experienced trauma, infection, or surgery 3 months before delivery; complications or complications during pregnancy, such as diabetes, hypertension, dangerous placenta previa, and intrahepatic cholestasis of pregnancy; severe fetal malformation; intrauterine pneumonia; diaphragmatic hernia; cardiogenic dyspnea; and congenital diseases.

### 2.2. Research methods

#### 2.2.1. Data collection

Data of newborns and their mothers with meconium-contaminated amniotic fluid were collected from the hospital’s obstetrics information system. Collected data included maternal age, reproductive history, gestational age, amniotic fluid index before delivery, delivery method, amniotic fluid contamination level, hydrogen ion concentration index (pH) and alkaline residue (BE) index in the umbilical cord blood at birth, newborn’s birth weight (weight of the newborn weighed for the first time within 1 hour of birth), newborn’s gender, and newborn’s Apgar score at 1 minute.

#### 2.2.2. Diagnosis and grouping of neonatal MAS

According to the Chinese Journal of Practical Neonatology (4th edition), the criteria for MAS include,^[9]^ meconium in the amniotic fluid and staining and yellowing of the umbilical cord, toenails, and skin of newborns; aspiration of meconium from the trachea of newborns; symptoms of respiratory distress; and widespread nodular patchy shadows on chest X-ray accompanied by emphysema or atelectasis. Newborns were divided into the MAS and non-MAS groups based on whether they developed MAS within 3 days of birth.

### 2.3. Statistical analysis

The SPSS19.0 statistical software, all data are presented as mean ± standard deviation. The t-test was used to compare data between the 2 groups. Categorical variables are expressed in frequency (n [%]) and compared using the *χ*^2^ test. Multiple logistic regression analysis was used to explore factors that influenced MAS development, *P *< .05 indicated statistical significance. A column chart prediction model for neonatal MAS risk was constructed using the R3.4.3 software. The performance of the model was evaluated using receiver operating characteristic (ROC) curve analysis and the Hosmer–Lemeshow goodness-of-fit test, and the clinical effectiveness of the model was evaluated using decision curves.

## 3. Results

### 3.1. Baseline characteristics of patients

**Figure 1.**
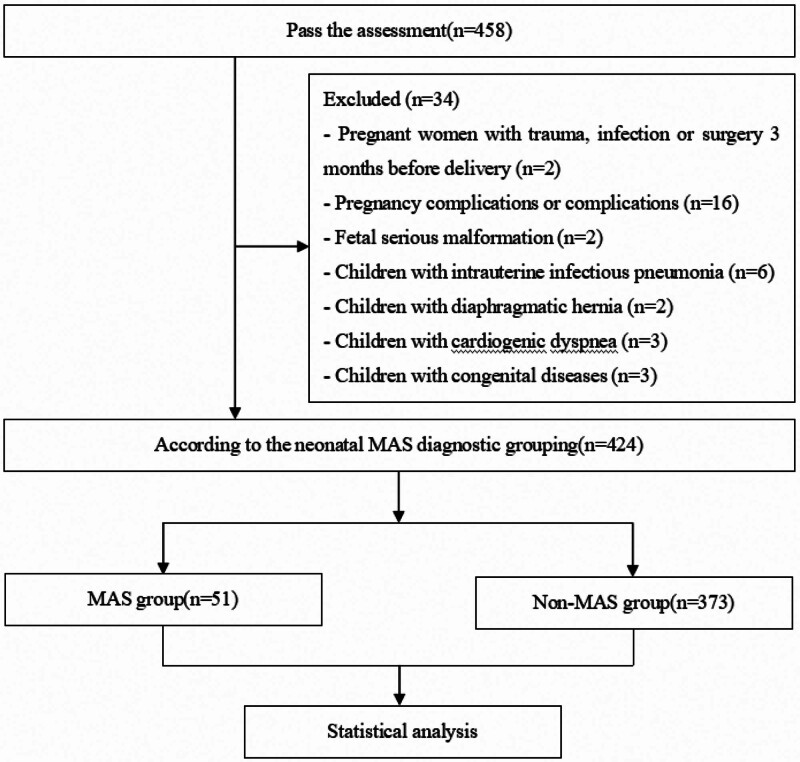
Research flowchart.

Of the 458 identified cases, 34 patients were excluded, and 424 patients ultimately participated in this study (Fig. 1). Overall, 51 newborns developed MAS within 3 days of birth, with an incidence rate of 12.03% (51/424). As shown in Table 1, gestational age, predelivery amniotic fluid index, delivery method, amniotic fluid contamination level, newborn umbilical cord blood pH, and newborn Apgar 1-minutes score were significantly different between the MAS and non-MAS groups (*P *< .05).

**Table 1:** Comparison of perinatal data between MAS and non-MAS groups.

Factor	MAS group (n = 51)	Non-MAS group (n = 373)	*χ^2^/t* value	*P* value
Maternal age (yr)	28.04 ± 2.78	27.54 ± 2.52	1.312	.190
Reproductive history			0.407	.524
Primipara	33 (64.71)	224 (60.05)		
Multipara	18 (35.29)	149 (39.95)		
Gestational age (wk)	39.58 ± 1.73	38.19 ± 1.15	7.551	<.001
Prepartum amniotic fluid index (cm)	7.04 ± 2.28	9.33 ± 2.31	6.650	<.001
Delivery method			4.942	.026
Cesarean birth	28 (54.90)	144 (38.61)		
Vaginal delivery	23 (45.10)	229 (61.39)		
Amniotic fluid pollution level			24.238	<.001
Grade I	1 (1.96)	85 (22.79)		
Grade II	19 (37.26)	179 (47.99)		
Grade III	31 (60.78)	109 (29.22)		
Newborn umbilical cord blood pH value	7.21 ± 0.09	7.38 ± 0.12	9.745	<.001
Neonatal umbilical cord blood BE value	−4.48 ± 1.24	−4.61 ± 1.29	0.678	.498
Newborn birth weight (kg)	3.25 ± 0.53	3.40 ± 0.66	1.556	.121
Newborn gender			0.271	.603
Male infant	30 (58.82)	205 (54.96)		
Female infant	21 (41.18)	168 (45.04)		
Neonatal Apgar 5-min score (points)	7.73 ± 1.52	8.56 ± 1.78	3.175	.002
Neonatal Apgar 5-min score (points)	9.34 ± 1.05	9.63 ± 1.25	1.582	.114

BE = alkaline residue, MAS = meconium aspiration syndrome, pH = hydrogen ion concentration index.

### 3.2. Multivariate logistic regression analysis of neonatal MAS

The occurrence of MAS in full-term newborns (0 = no; 1 = yes) was used as the dependent variable, and *P *< .05 was used as the independent variable (Table 1). Multivariate logistic regression analysis showed that a low amniotic fluid index before delivery, advanced gestational age, cesarean section, severe amniotic fluid contamination, low neonatal umbilical cord blood pH, and low neonatal Apgar 1-min score were factors influencing the development of neonatal MAS in full-term newborns (*P *< .05) (Table 2).

**Table 2:** Results of multivariate analysis of neonatal MAS.

Variable	*β*	*SE*	Waldχ^2^	*P* value	*OR*(95%*CI*)
Low amniotic fluid index before delivery	1.051	0.448	5.504	.019	2.862 (1.189~6.883)
Gestational age	−0.642	0.303	4.489	.034	0.526 (0.291~0.953)
Cesarean birth	0.975	0.392	6.186	.013	2.650 (1.230~5.714)
Amniotic fluid pollution level	1.435	0.464	9.565	.002	4.199 (1.692~10.423)
Newborn umbilical cord blood pH value	1.078	0.424	6.464	.011	2.938 (1.280~6.746)
Newborn Apgar 1-minute score	1.142	0.416	7.536	.006	3.133 (1.387~7.078)

MAS = meconium aspiration syndrome, pH = hydrogen ion concentration index.

### 3.3. Construction of a column chart prediction model for the risk of MAS in full-term newborns

**Figure 2.**
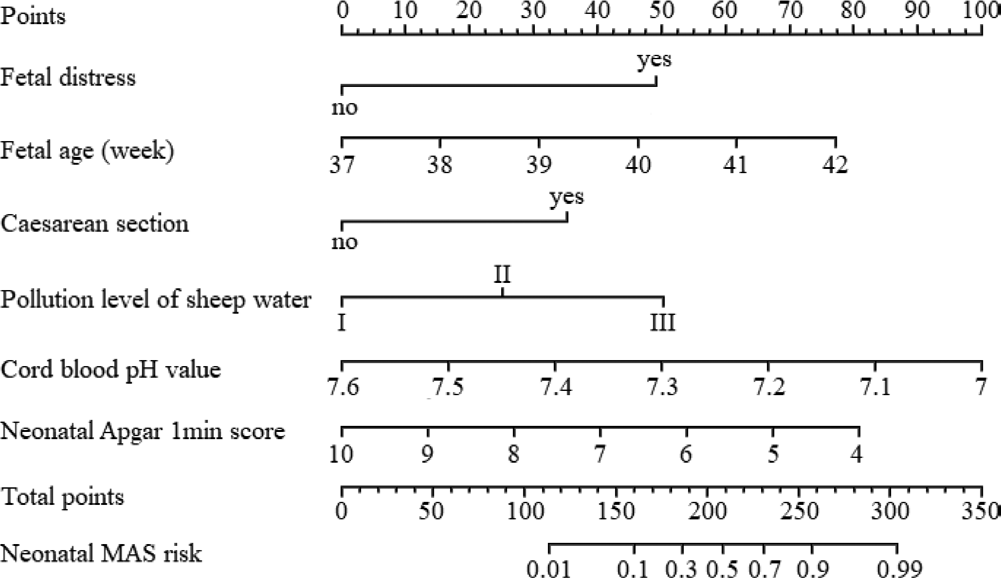
Nomogram prediction model for neonatal MAS risk. MAS = meconium aspiration syndrome.

A predictive model expressed in a column chart was constructed based on the 6 influencing factors selected through multiple logistic regression analysis. As shown in Figure 2, by presenting the corresponding scores for each factor value through a graphical representation, the total score was obtained by adding the scores for each factor and converting the total score into the predicted probability of risk.

### 3.4. Performance analysis of the column chart prediction model for the risk of MAS in full-term newborns

**Figure 3.**
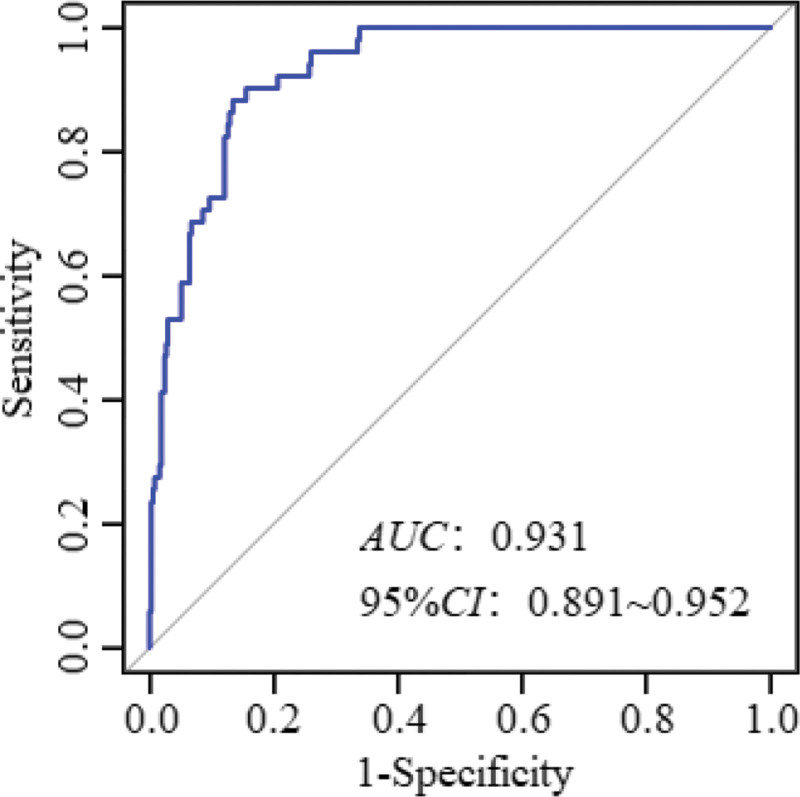
Column chart model of the *ROC* curve evaluation of neonatal MAS risk. MAS = meconium aspiration syndrome, ROC = receiver operating characteristic.

**Figure 4.**
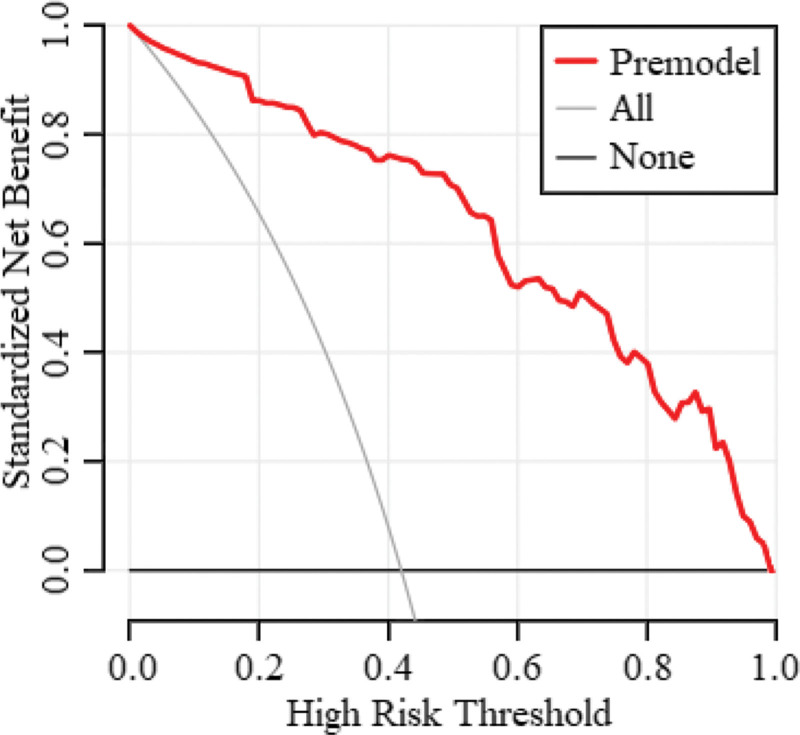
Evaluation of the decision curve of the column chart model for neonatal MAS risk. MAS = meconium aspiration syndrome.

According to the ROC curve analysis, the area under the ROC curve of the nomogram expression model in predicting the risk of MAS in full-term newborns was 0.931 (95% confidence interval [CI]: 0.891–0.952), and the sensitivity and specificity were 0.882 and 0.866, respectively (Fig. 3). The goodness-of-fit bias of the model was *χ*^2^ = 3.465 and *P *= .903, and the model had a good fit. Decision curve analysis showed that the net benefit for newborns with meconium-contaminated amniotic fluid was higher than that of the other 2 extreme curves, indicating that the model had clinical validity (Fig. 4).

## 4. Discussion

The etiology of neonatal MAS is complex and characterized by the rapid onset and progression of the disease.^[10]^ Once MAS occurs, it can easily lead to difficulty breathing in newborns.^[11]^ Neonatal MAS is associated with several factors and can lead to recurrent and persistent hypoxia in newborns, inducing multiple organ damage^[1]^ and reducing quality of life. Therefore, early and effective identification of newborns who may develop MAS is beneficial for adopting targeted measures to reduce or avoid the harm caused by MAS.

Oligohydramnios can rapidly lead to fetal distress, causing ischemia and hypoxia in the uterus. Rabie et al reported that oligohydramnios during pregnancy increases the risk of neonatal MAS,^[12]^ consistent with the results of this study. This may be due to oligohydramnios causing intrauterine hypoxia, which mediates the relaxation of the fetal rectal sphincter that causes the discharge of meconium into the amniotic fluid,^[13]^ and simultaneously stimulating the fetal respiratory center to induce wheezing.^[14]^ Inhalation of meconium and amniotic fluid can easily induce MAS.

Ward et al reported that the risk of MAS increases with an increase in term gestational age,^[15]^ which is consistent with the results of this study. The placenta has the function of clearing meconium. As the gestational age increases, the placenta begins to show varying degrees of calcification and aging, causing a decrease in placental function. Thus, it is unable to effectively remove meconium, increasing the risk of amniotic fluid contamination with meconium and thereby increasing the risk of MAS in newborns.

Chand et al reported that cesarean section was an influencing factor of MAS in term infants,^[16]^ which is consistent with the results of this study. Cesarean section avoids compression of the fetal head during vaginal delivery. When the fetus is in the mother’s body, the lungs are filled with fluid; if the head is compressed in the birth canal during vaginal delivery, it is possible to expel a portion (approximately 1/3–1/2) of the lung fluid through the mouth and nose, and the remaining part is absorbed by pulmonary interstitial capillaries and lymphatic vessels after birth.^[17]^ Therefore, for fetuses with meconium-contaminated amniotic fluid who have already inhaled meconium, the lack of measures to promote lung fluid discharge through the compression of the birth canal, failure to clean the respiratory tract in a timely manner, or incomplete cleaning after birth increases the risk of MAS following cesarean section.

The more severe the meconium contamination of amniotic fluid, the higher the probability of fetal hypoxia, and the more likely MAS is to occur.^[18]^ The cause of suffocation is hypoxia. Moreover, fetal asphyxia is associated with intrauterine distress. Hypoxia can not only cause the fetus to easily excrete meconium, which further contaminates amniotic fluid, but also aggravate fetal wheezing, inducing fetal deep breathing that increases the probability of MAS occurring due to inhalation of fecal-stained amniotic fluid. The Apgar score is reportedly associated with the degree of amniotic fluid pollution.^[19]^ Oliveira et al reported that a low Apgar 1-min score is a risk factor for the development of neonatal MAS,^[20]^ which is consistent with the results of this study. The more severe the contamination of amniotic fluid by meconium, the lower the Apgar score, the more severe the suffocation, and the more likely the development of various complications.

The perinatal stress status of newborns can be determined by umbilical artery blood gas analysis. Blackwell et al found that the occurrence of MAS is closely related to the acid-base status of the umbilical cord blood during delivery.^[21]^ This may be due to an acid-base imbalance in the umbilical blood, indicating excessive acidic substances in the fetal body that easily induce acidosis. The pH and BE values of umbilical cord blood are commonly used to reflect the acid-base balance. Choi et al reported that a low umbilical cord blood pH was a risk factor for MAS in neonates,^[22]^ which is consistent with the results of this study. An abnormal acid-base status in cord blood, which represents an increase in acidic substances in the fetal body, may easily cause inflammatory exudation, enhance the inflammatory response,^[23,24]^ and stimulate the fetus to breathe autonomously. Amniotic fluid contaminated by meconium in the oropharynx gradually migrates to the small airway, causing obstruction, excessive inflation, or atelectasis of the lungs that increases the risk of MAS.

We also constructed a nomogram prediction model based on the predelivery amniotic fluid index, gestational age, cesarean section, degree of amniotic fluid contamination, low newborn cord blood pH, and low newborn Apgar 1-min score. According to the *ROC* curve analysis, the area under the ROC curve value of the nomogram expression model in predicting the risk of neonatal MAS in full-term pregnant women was good, which shows that the model has a high prediction ability. This may be related to the full consideration of the interactions between the factors in this study by combining multiple indicators for prediction, thereby effectively improving the predictive performance of the model. The decision curve analysis showed that the net benefit of the model in predicting the risk of MAS in newborns with meconium-contaminated amniotic fluid was higher than that of the other 2 extreme curves, indicating the clinical validity of the model. This also further proves that a low amniotic fluid index before delivery, advanced gestational age, cesarean section, amniotic fluid contamination, low pH value of neonatal umbilical cord blood, and low Apgar 1-minute score influence the development of neonatal MAS. Therefore, combining these factors into a prediction model to predict the risk of neonatal MAS in advance is beneficial for clinicians to adopt targeted measures to prevent and reduce the risk of neonatal MAS, which is more meaningful than initiating treatment after the occurrence of neonatal MAS.

This study had limitations. First, this was a single-center retrospective analysis with a limited sample size, which may have affected the results. Second, the predictive model constructed in this study has not undergone external or prospective validation, and its reliability requires further evidence. Therefore, in the future, multicenter research and expansion of the sample size are needed to further verify the reliability of the model and provide more reliable evidence for clinical research.

## 5. Conclusion

In conclusion, a low amniotic fluid index, gestational age at delivery, cesarean section, degree of amniotic fluid contamination, low neonatal umbilical cord blood pH value, and low neonatal Apgar 1-min score were associated with MAS in neonates. Creating a column chart prediction model based on the above indicators can provide guidance for clinical medical personnel to assess the risk of neonatal MAS early and formulate preventive strategies.

## Author contributions

**Conceptualization:** Chen Ling.

**Data curation:** Chun-Yu Wang, Chen Ling, Li-Sha Guan, Xiao-Qing Wang.

**Funding acquisition:** Juan-Juan Yang, Li-Sha Guan, Xiao-Qing Wang.

**Investigation:** Juan-Juan Yang.

**Formal analysis:** Chun-Yu Wang, Chen Ling.

**Methodology:** Chun-Yu Wang, Juan-Juan Yang, Li-Sha Guan.

**Project administration:** Chen Ling.

**Software:** Li-Sha Guan.

**Supervision:** Chen Ling, Juan-Juan Yang, Xiao-Qing Wang.

**Validation:** Chen Ling, Juan-Juan Yang.

**Visualization:** Chen Ling, Juan-Juan Yang.

**Writing – original draft:** Chun-Yu Wang, Chen Ling.

**Writing – review & editing:** Chun-Yu Wang, Chen Ling.
